# A Great Wrong Righted

**Published:** 1882-12

**Authors:** 


### A GREAT WRONG RIGHTED.

Early in 1882, there appeared in the pages of the *American Medical Weekly*, edited by Dr. E. S. Gaillard, a long article from the pen of Dr. Ephraim Cutter on the subject of prepared food. Dr. Cutter claimed to be able to discover, by the use of the microscope, the percentages of the chief constituents of cereal flours and foods. He stated that he had microscopically examined over forty specimens of advertised foods, and gave cuts which purported to be made from drawings copied from his microscopic enlargements. He very earnestly asserted the infallibility of his microscope in determining the amount of starch and the amount of gluten in a specimen of flour, and his claim in this regard, as well as his conclusions in the cases presented, were warmly indorsed by Dr. Gaillard.

In the Cutter article nearly every food mentioned was passed over with a brief comment, generally of a rather non-committal character, a pleasant little compliment being vouchsafed to one or two, and mild reproach being bestowed upon others. When the "flour of the entire wheat," made by the Franklin Mills, of Lockport, N. Y., was reached, the vocabulary of adulation was all too limited for the expression of Dr. Cutter's ecstacy. Eureka! He had found, at last, the food which was destined to be the true savior of the race! Here was, indeed, "a blessing to mankind." In the midst of many frauds, surrounded by a wilderness of feeble foods, his unerring microscope had discovered the one great and good substance, the one much-needed preparation, "a reliable infant's food." He had "analyzed" it with his microscope; he knew exactly what it contained, and he gave two pictures to prove it, one of which was entirely full of what purported to be "gluten cells."

But clouds will come as well as sunshine, and sorrow is still the lot of mortals. The joy which pervaded Dr. Cutter's being at his grand discovery of mankind's Lockport blessing, was speedily turned to sorrow as he peered through his instrument at other samples of flour. He started back aghast at the terrible revelation! Here were wheat flours containing no trace of gluten! Here were powdered substances marked "gluten" which were absolutely innocent of that albuminoid! What wicked fraud was this which was being perpetrated upon suffering humanity? Who were the authors of this dire iniquity, by which the lives of young and old were put in daily jeopardy?

The chief offender was said to be the Health Food Company, of No. 74 Fourth avenue, New York. The microscopist could find nothing desirable in any of its products. The pictures assigned to it were dreary deserts of blank space, sprinkled here and there with unrecognizable dots—an open sea of nothingness specked with minute islands called "starch granules." The saddest thought of all was, that an establishment organized for the ostensible purpose of providing better foods for the people, for the sick and the well alike, for the tender infant and the delicate invalid, for the weak who would be strong and the strong who would be stronger; that this institution, trusted by all, commended by physicians, presided over by an earnest physiologist, a chemist of practical ability, a microscopist of large experience—in short, that this prominent, able, intelligent, wealthy and prosperous house, widely known and highly esteemed, numbering among its patrons the President and thousands of the leading citizens of the United States, as well as the Royal Family of England—should become, whether innocently or willingly, the parties to the frauds charged upon them by Dr. Ephraim Cutter and his editor, Dr. E. S. Gaillard!

It is difficult to find a single physician now who is willing to admit that he believed Dr. Cutter's statements, or that he failed to quickly discover their absurdity. But it is evident that worthy men were deceived by them. Cutter announced his name and titles—such of them as he could remember—with a fine flourish, and those who read the schedule for the first time, naturally supposed that he was a real authority upon matters pertaining to microscopy. It may be that Dr. Gaillard was one of these. Dr. Abram Jacobi evidently was, for he adopted and commended Cutter's statements. If that astute medical man could be thus deceived, it is clear that nothing better could be expected from the public at large.

Some prominent medical gentlemen detected the fallacies of Cutter, and exploded them—Dr. George B. Fowler, of New York, and Prof. Joseph G. Richardson, of the University of Pennsylvania, among the rest. But it remained for Dr. Albert R. Leeds, Professor of Chemistry in the Stevens Institute of Technology, and Public Analyst for the State of New Jersey, to completely refute the statements of Cutter, whether those statements were commendatory or condemnatory. Prof. Leeds took up the foods named by Cutter, one by one, first carefully examining them microscopically and afterward subjecting them to rigid chemical analysis. He found the microscope to be valueless as a means of determining the percentages of gluten and starch in pulverized grain, and was forced to rely for accurate knowledge upon chemical analysis. Exhaustive labor in this direction resulted in conclusively proving that Cutter's assertions were untrue, and that what he claimed as honest revelations of the microscope could have been nothing more valuable than figments of the imagination, even if they did not deserve a harsher term. Prof. Leeds showed, moreover, some curious things. He found that the poorest wheat flour attainable was one which Dr. Cutter stated, as the result of his alleged microscopical inquiry, was "rich in gluten and in all the elements of entire wheat." It proved to be made in Massachusetts by a Mr. Fowler, who is a brother-in-law of Cutter! It is called "Arlington meal."

Another dangerous misstatement is shown by Prof. Leeds by his analysis of the Lockport flour—the "Franklin Mills flour of the entire wheat," so called—so enthusiastically extolled by Dr. Cutter. Prof. Leeds finds this "blessing to mankind" to rank below the minimum in gluten, to be, in fact, less nutritive than common white flour. This fact is so important, in view of the exalted claims put forth by the manufacturers of this flour, and of the extravagant praise uttered by Cutter, that we deem it proper to copy a few lines from Prof. Leeds' Report. Speaking of the Lockport article, he says: "The sample which I analyzed, and which I purchased myself from the accredited agent of the 'Franklin flour,' in New York, contained an unusually low percentage of nitrogenous substance. It yielded on analysis but 8.55 per cent. of albuminoids, which is less than the *minimum* amount found as above quoted by Prof. Kedzie, in 16 analyses of winter wheat flour, and four per cent. less than the average amount in five samples of Kansas spring wheat flour. Its percentage of starch was 65.23 per cent. In other words, this flour, which contained so many gluten cells that the field of the microscope, according to Dr. Cutter, in one instance, was entirely filled with them, contained several per cent. less of gluten than ordinary flour, and but little less than the usual amount of starch. What made up the deficit? It was cellulose, and it is to the bran that the dark color of the bread made from this flour is due. But when, as in this case, the bran is accompanied by even less gluten than in common flour, the bread prepared from flour so made is inferior in nutritive value to the bread made from common flour, and is inferior also in sweetness, taste and appearance."—("*Scientific Examination of Foods*," *page* 8.)

Prof. Leeds finds in the whole wheat flour of the Health Food Company 16.74 per cent. of albuminoids, usually termed gluten, and says concerning it, "It is thus proved to be 8.19 per cent. richer in gluten than the Franklin Mills flour of the entire wheat, so greatly admired by Dr. Cutter; in other words, it is almost twice as valuable as food." He proves by comparative analysis, that the gluten flour of the Health Food Company is purer than that made by Conor in Paris, and adds: "So far as I know, no more highly nitrogenous flours exist than those made by this company. I have encountered nothing, therefore, more valuable with which to compare them." Prof. Leeds concludes as follows:

"* * * * * I am persuaded that Dr. Cutter's methods of microscopic inquiry have led him to commit mistakes, calculated to do great injury to many reputable business men. Certainly, in the case of the Health Food Company * * * his results are entirely false. And his errors are rendered the less accountable from the fact that he has condemned some of these products as worthless, or worse, on account of containing no gluten, when in truth they contain 50 per cent. more of gluten than the Franklin flour or the Arlington meal with which they are contrasted. What an inexcusable injury to the manufacturers of many excellent infant foods, when he has condemned them as injurious to infants on the ground of their containing a great deal of starch and no gluten, while he has extolled as a "reliable infants' food" the Franklin flour, which contains less albuminoids than any wheat product I have thus far examined, except the Arlington wheat meal! * * I think it is but just to the many manufacturers whom his article on cereal foods has greviously wronged, to condemn it as a false and mischievous publication. It has placed Dr. Cutter in the ranks of those writers on the subject of food, who by statements easily recognizable as untrue by any informed person, and frequently at variance with common sense, have done so much to bring the subject of the adulteration of food into the category of sensational pseudo-scientific literature."

In dismissing the subject for the present, we leave it to the intelligence of our readers to judge of the motive which actuated Dr. Cutter and others concerned in this libelous attack upon the Health Food Company and its valuable products. That it was intended to signally injure them while greatly benefitting another, is evident upon its face. That there was something of malice in it, is not improbable. We know that there is an inside history to it not known to the public. We know that the head of the Health Food Company several years ago bargained for the Franklin mills at Lockport, New York, with Mrs. Hunt, their owner, and Senator St. John, of Newburg, her attorney. We know that the late Governor Hunt was the warm personal friend of the head of the Health Food Company —both being Ex-Governors—and that the bargain which he was enabled to make at Newburgh was a most favorable one. We have been informed that the advantages anticipated from that purchase were lost to him by reason of the unfaithfulness of one whom he trusted, and whom he empowered to act in his behalf. We know, too, that he believes the man who thus wronged him to be capable of conspiring to do him this later injustice, now so signally rebuked by unerring science. We are told that to every physician in the country a free copy of *Gaillard's American Medical Weekly* containing the libel, was sent. To such of them as may chance to read these words, we beg to suggest that they address a line to Prof. A. R. Leeds, Stevens' Institute, Hoboken, New Jersey, and ask for a copy of the report to which we have alluded. They will be richly repaid by its perusal, and will rejoice with us in the effectual righting of a great wrong.

